# Rapid and Accurate Diagnosis of Dermatophyte Infections Using the DendrisCHIP^®^ Technology

**DOI:** 10.3390/diagnostics13223430

**Published:** 2023-11-11

**Authors:** Aurore Anton, Mathilde Plinet, Thomas Peyret, Thomas Cazaudarré, Stéphanie Pesant, Yannick Rouquet, Marie-Andrée Tricoteaux, Matthieu Bernier, Jérémy Bayette, Remi Fournier, Mélanie Marguerettaz, Pierre Rolland, Thibaud Bayol, Nadia Abbaoui, Antoine Berry, Xavier Iriart, Sophie Cassaing, Pamela Chauvin, Elodie Bernard, Richard Fabre, Jean-Marie François

**Affiliations:** 1 Dendris SAS, 335 Rue du Chêne Vert, 31670 Labège, France; mplinet@dendris.fr (M.P.); tpeyret@dendris.fr (T.P.); tcazaudarre@dendris.fr (T.C.); pesant.s@chu-toulouse.fr (S.P.); ebernard@dendris.fr (E.B.); rfabre@dendris.fr (R.F.); fran_jm@insa-toulouse.fr (J.-M.F.); 2 Laboratoire Inovie-CBM, 31000 Toulouse, France; yannick.rouquet@inovie.fr (Y.R.); marie-andree.tricoteaux@inovie.fr (M.-A.T.); matthieu.bernier@inovie.fr (M.B.); 3 Laboratoire Inovie-Labosud, 34070 Montpellier, France; jeremy.bayette@inovie.fr (J.B.); remi.fournier@inovie.fr (R.F.); melanie.marguerettaz@inovie.fr (M.M.); pierre.rolland@inovie.fr (P.R.); thibaud.bayol@inovie.fr (T.B.); nadia.abbaoui@inovie.fr (N.A.); 4 Service de Parasitologie-Mycologie, Centre Hospitalier Universitaire Purpan de Toulouse, Institut Fédératif de biologie (IFB), 31300 Toulouse, France; berry.a@chu-toulouse.fr (A.B.); iriart.x@chu-toulouse.fr (X.I.); cassaing.s@chu-toulouse.fr (S.C.); chauvin.p@chu-toulouse.fr (P.C.); 5 Institut Toulousain des Maladies Infectieuses et Inflammatoires (Infinity), Hôpital Purpan, 31024 Toulouse, France; 6 Toulouse Biotechnology Institute (TBI), Université de Toulouse, Institut National des Sciences (INSA), 135 Avenue de Rangueil, 31077 Toulouse, France

**Keywords:** dermatophytes, in vitro diagnostic, syndromic approach, multiplex PCR, biochips, microbial cultures, machine-learning methods

## Abstract

Dermatophytosis is a superficial fungal infection with an ever-increasing number of patients. Culture-based mycology remains the most commonly used diagnosis, but it takes around four weeks to identify the causative agent. Therefore, routine clinical laboratories need rapid, high throughput, and accurate species-specific analytical methods for diagnosis and therapeutic management. Based on these requirements, we investigated the feasibility of DendrisCHIP^®^ technology as an innovative molecular diagnostic method for the identification of a subset of 13 pathogens potentially responsible for dermatophytosis infections in clinical samples. This technology is based on DNA microarray, which potentially enables the detection and discrimination of several germs in a single sample. A major originality of DendrisCHIP^®^ technology is the use of a decision algorithm for probability presence or absence of pathogens based on machine learning methods. In this study, the diagnosis of dermatophyte infection was carried out on more than 284 isolates by conventional microbial culture and DendrisCHIP^®^DP, which correspond to the DendrisCHIP^®^ carrying oligoprobes of the targeted pathogens implicated in dermatophytosis. While convergence ranging from 75 to 86% depending on the sampling procedure was obtained with both methods, the DendrisCHIP^®^DP proved to identify more isolates with pathogens that escaped the culture method. These results were confirmed at 86% by a third method, which was either a specific RT-PCR or genome sequencing. In addition, diagnostic results with DendrisCHIP^®^DP can be obtained within a day. This faster and more accurate identification of fungal pathogens with DendrisCHIP^®^DP enables the clinician to quickly and successfully implement appropriate antifungal treatment to prevent the spread and elimination of dermatophyte infection. Taken together, these results demonstrate that this technology is a very promising method for routine diagnosis of dermatophytosis.

## 1. Introduction

The global prevalence of superficial fungal infections affects around 20 to 25% of the world’s population [1], with dermatophytosis being the most common mycosis. An estimated 10 to 15% of human beings are infected at some point in their lives by dermatophytes [2], making this infection a major public health problem [3]. This type of infection is mainly attributed to five or six species of dermatophytes, which are filamentous fungi with the ability to invade keratinized tissues, such as skin, hair, and nails [4,5]. Even if *Trichophyton rubrum* is the most common pathogen associated with ringworm or tinea infections, dermatophytosis can also be caused by opportunistic pathogens such as yeast or non-dermatophyte molds [6].

Conventional dermatophyte diagnostics rely on microbiological and biochemical methods. Currently, the gold standard for dermatophyte identification consists of microscopic examination of clinical specimens followed by culture to identify the specific type of fungus. While not materially expensive, this protocol is time-consuming, requiring 10 to 15 days or even up to 3 to 4 weeks to provide results due to the long incubation period for growth. In addition, the diagnostic analysis often depends on the expertise of the clinical staff and requires highly skilled operators [7]. 

Clinical laboratories, therefore, urgently need faster and unbiased diagnostic methods to overcome these limitations, as rapid identification of the causative agent will enable appropriate antifungal treatment. In addition, these new methods will make it possible to track sources of infection and help manage outbreaks to avoid reinfection [8]. In this regard, molecular tests based on PCR methodology have been developed for the detection/identification of dermatophytosis over the last decade [9,10]. These include multiplex PCR with species identification via product length visualized on agarose-gel [11], multiplex PCR with pan-dermatophyte primers combined with species-specific primers for *T. rubrum* [12], pan-dermatophyte nested PCR [13,14], real-time PCR using sets of species-specific primers and probes [15,16,17,18,19], and PCR-RFLP [20,21], PCR-RLB [22], and PCR-ELISA tests [23,24]. Another currently used technique for identifying microorganisms is the analysis of protein profiles by MALDI-TOF MS, which, however, requires pure cultures [25]. Although these methods are attractive due to their speed, sensitivity, and reproducibility, they are restricted to the identification of a few species per sample/patient due either to limited multiplexing capabilities or the technical limits of proteomics. In addition, these methods also require technological expertise that is often not available in clinical laboratories.

In this study, we present a new rapid and accurate method to diagnose dermatophyte infections that relies on DendrisCHIP^®^ technology. Although DNA microarray technology has already been proposed as a method for identifying pathogens in various clinical contexts [26,27], this technology has remained remote from field applications, particularly in clinical microbiology, due to its relatively low sensitivity and/or specificity, the complexity of the biochip manufacturing process and finally, the difficulty of providing the diagnostic results. These limitations have been, to a great extent, overcome by the DendrisCHIP^®^ technology [28]. This technology features two major innovations: (i) the DendriSLIDE, a glass slide functionalized by dendrimer chemistry which greatly improves the signal-to-noise ratio between oligonucleotide probes and the nucleic acid target, thereby increasing the sensitivity of the method [29,30], and (ii) the application of machine learning methods that rapidly provide the diagnosis in the form of a probability value for the presence or absence of pathogens in the sample, as well as discriminating the pathogen(s) at the genus or species level involved in the infection. Overall, DendrisCHIP^®^ technology provides a diagnostic result within 48 h after sample receipt, which is comparable to multiplex PCR technologies and much faster than conventional cultures. This technology has proven to be technically reliable for the diagnosis of respiratory infectious diseases [28] and osteoarticular infections [29]. The present report deals with the demonstration that this technology is also well suited for rapid and accurate diagnosis of dermatophyte infections.

## 2. Materials and Methods

### 2.1. Microbial Strains

Pure microbial strains listed in Table 1 were purchased from the Belgian Coordinated Collections of Microorganisms-Scientific Institute of Public Health (BCCM/IHEM, Belgium), the Colección Española de Cultivos Tipo (CECT, Valencia, Spain), and Department of Parasitology-Mycology of the Centre Hospitalier Universitaire de Toulouse Hôpital Purpan (CHU Purpan, Toulouse, France), Institut Fédératif de Biologie (IFB, Toulouse, France).

### 2.2. Clinical Specimen’s Provision

Swabs, skin scrapings, nails, and hair samples from patients were collected and provided by independent clinical laboratories of Toulouse and Montpellier (INOVIE Group). A total of 284 samples were simultaneously analyzed by conventional mycological cultures by these laboratories according to their internal practices and by DendrisCHIP^®^ technology at DENDRIS laboratory. Of these, 197 were skin scrapings, nails, and hair samples, and 87 were swabs rubbed onto patients’ lesions after conventional sampling.

### 2.3. DNA Extraction and PCR Amplification

DNA extraction from isolates requires pre-treatment with the DendrisKIT^®^DP. Solid specimens were placed in 400 µL DLB solution, whereas swab samples required strong shaking on vortex to obtain a homogenous suspension after being soaked in 400 µL DLB solution. Then, 10 µL of DPK solution was added, and the suspensions were incubated at room temperature overnight. DNA extraction and purification from these suspensions were then carried out using DNeasy Blood & Tissue Kit from Qiagen (Qiagen, Courtaboeuf, France) according to the manufacturer’s instructions.

DendrisKIT^®^DP also contains DendrisPCR DP, corresponding to a mixture of 7 fungus-specific primers used to amplify the full ITS sequence, including the 5.8S rRNA gene. Reverse primers are labeled at the 5′ end with biotin (bio-teg). PCR reaction was performed with 5 µL of template DNA in a total reaction volume of 50 µL with TaKaRa Taq™ DNA Polymerase Hot Start Kit (TaKaRa Bio group, Europe) with each primer between 0.05 and 0.2 µM (Integrated DNA Technologies, Clareville, IA, USA). The amplification was achieved with a PCR in a Rotor-Gene Q (Qiagen, Nehren, Germany) using the following program: 94 °C for 3 min; 40 cycles of denaturation (94 °C, 1 min), annealing/extension (60 °C, 1 min); and a final extension at 72 °C for 1 min.

### 2.4. Design of Oligonucleotide Probes

A total of 44 probes were designed on the hypervariable region of the ITS rRNA sequence from the pathogens listed in Table 1. Multiple alignment analysis using ClustalW (http://www.clustal.org/clustal2/, accessed on 30 November 2021) was applied to the ITS rRNA sequence, which was retrieved from the NCBI database or sequenced prior to creating the probe design (see below). The exclusivity of the probe sequence was queried against sequences in Genbank database with a BLAST search. Probe quality criteria, namely, length of the oligonucleotide between 20 and 25 nucleotides, equal melting temperature, lack of hairpin, and dimer formation, were assessed with Primer 3plus [30]. Synthetic oligoprobes were further designed for quality control of the process. The probes were purchased from Integrated DNA Technologies (IDT, Leuven, Belgique) with their 5′ ends NH2-modified.

### 2.5. Manufacture of the DendrisCHIP^®^DP

The DendrisCHIP^®^ bearing the oligoprobes of the targeted pathogens implicated in dermatophytosis was termed DendrisCHIP^®^DP. They were manufactured using oligoprobes at a concentration of 50 µM in 0.2 M phosphate buffer pH 9.0 and printed in triplicate on DendriSLIDE by piezo electrical dispensing with the sci-FLEXARRAYER SX robot from Scienion (Berlin, Germany). The average diameter of spots was 180 µm ± 10 µm and each spot was spaced by 350 µm. Two additional oligoprobes were included in this pattern for quality control (QC) purposes. The first, “REF”, was a 25-mer synthetic probe enabling the positioning of the DendrisCHIP^®^DP during the image processing. The second, “CIH_ol”, was a 25-mer synthetic probe for validation of the hybridization step. At the end of the spotting process, the DendrisCHIP^®^DP were plunged in a bath containing 1.74 g/L of NaBH4 in milli-Q water for 30 min followed by three washes in water for 5 min each before being dried by centrifugation at 900 rpm for 30 min. Then, a custom plastic structure (MPM, Muret, France) is placed over each slide, separating it into sixteen independent units for analysis.

### 2.6. Hybridization Process and Reading of the DendrisCHIP^®^DP

PCR purification and hybridization of the purified amplicons on the DendrisCHIP^®^DP were performed in an automatic manner using the DendriSTATION. A Microlab Starlet (Hamilton, Bonaduz, Switzerland) controlled by VENUS 4.0 equipped with a Hamilton HeaterShaker (HHS, Hamilton, ON, Canada), a Hamilton Heater cooler (HHC, Hamilton, ON, Canada), a HeatPAC ambient 135 °C (Inheco, Planegg, Germany), a NucleoMag SEP (Macherey-Nagel, Düren, Germany), and an HEPA hood (Noroit, Bouaye, France). The practical condition for hybridization was as follows: heating PCR amplicons (50 µL) for 2 min at 95 °C and quenching at 4 °C followed by addition of 110 µL of a hybridization buffer 50X Denhardt’s solution (with 1% Ficoll (type 400), 1% polyvinylpyrrolidone and 1% Bovine Serum Albumin), 2.5× SSC, 100 µg/mL salmon sperm DNA, and the 5′bioteg-labelled oligonucleotide complementary of CIH_ol at a final concentration of 1 nM. Then, 125 µL of this mix was pipetted and loaded in each well of DendrisCHIP^®^DP. The chips were then incubated at 60 °C on a shaker set at 250 rpm for 30 min. The mix was aspirated, and wells were washed once with 200 µL of washing buffer 1 (1× PBS). Then, 100 µL of diluted HRP-streptavidin was added and incubated in the dark for 20 min. The solution was aspirated, and wells were washed three times with 200 µL of washing buffer 2 (1× PBS, 0.05% Tween-20). In the last step, 100 µL of sciCOLOR T3 substrate (Scienion, Berlin, Germany) was added into the wells and incubated again for 20 min in the dark. The substrate was removed, and the chips were dried at 50 °C for 5 min and then left at room temperature for another 5 min. The DendrisCHIP^®^DP were read using the DendriSCAN sciREADER CL2 (Scienion Inc., Berlin, Germany) reader. Results were compiled in xlsx spreadsheets.

### 2.7. Data Treatment Using Machine-Learning Methods and Statistical Analysis

The first step to exploiting the machine learning method is to build a training set. Here, it consists of hybridization signals obtained from 441 samples from pure strains, mixed strains, and isolates with known microorganisms, as well as spiked with pure fungi strains. In this training set, the targets to be predicted are those presented in Table 1 (*n* = 12), and the variables are the designed probe hybridization signals. Of note, a target can be ascribed as a family/genus or species. Thus, species belonging to a family/genus were labeled for both the family/genus and the species (e.g., *Trichophyton rubrum* must be detected as *Trichophyton rubrum* and *Trichophyton* spp. simultaneously). After a comparative study of the learning algorithms, the hierarchy of multilabel classifier (HOMER) [31] combined with the multilabel version of the random forest proposed by Abarenkov et al. [32] turned out to be the best performing. This version is implemented in the R package utiml [33], using the function homer from utiml with default parameters for clustering (clusters with K = 3, method = balanced) and with parameter “base.algorithm” RF for the learning. The prediction was computed with the function predict. Then, to determine algorithm performance, cross validation was performed while using the leave-one-data-point-out (LOOCV) method on the database. The function multilabel_evaluate with parameter measure = “all” was used to obtain the accuracy, F1 score, and hamming loss. The confusion matrix was computed to obtain true positives (TP), true negatives (TN), false positives (FP), and false negatives (FN) with the function multilabel_confusion_matrix. 

### 2.8. Assessment of the Limit of Detection (LoD)

To assess the limit of detection (LoD) of the technology, PCR was performed on serial dilution of pure genomic DNA from each pathogen, and the labeled amplified targets were hybridized on DendrisCHIP^®^DP. To obtain statistically significant data, this experiment was repeated 10 times by serial dilutions.

### 2.9. Diagnosis by Microbiological Cultures 

In two independent clinical laboratories, diagnosis was established based on mycological examination techniques as accredited by COFRAC (Number 8-3580). All specimens were cultured on two plates/tubes, one containing Sabouraud dextrose agar (SDA) supplemented with gentamicin (Bio-Rad, Marnes-la-Coquette, France) and the other one containing SDA supplemented with gentamicin plus actidione (Bio-Rad, Marnes-la-Coquette, France) for fungal identification. Cultures were incubated at 25–30 °C and evaluated for growth after 48 h and then once daily for a month. Positive specimens for dermatophytes were identified according to three criteria: growth rate, macroscopic, and microscopic characteristics of colonies. Part of the sample was stored at ambient temperature and used for molecular diagnosis, including PCR, NGS, and DendrisCHIP^®^ technologies.

### 2.10. Diagnostic Analysis Using RT-PCR and NGS

RT-PCR was performed on the CFX-96 (Bio-Rad, Paris, France) using the DermaGenius^®^ 2.0 Complete multiplex kit (PathoNostics, Maastricht, The Netherlands) according to the manufacturer’s instructions. Reagents for performing two separate multiplex PCR procedures were used: Master Mix 1 (MMX1) contained the originally designed specific PCR primer pairs and detection probes for *C. albicans*, *T. interdigitale*, *T. mentagrophytes*, *T. rubrum/soudanense*, *T. tonsurans* and *T. violaceum*, and MMX2 contained the originally designed primer pairs and probes for *E. floccosum, M. audouinii*, *M. canis*, *T. benhamiae*, and *T. verrucosum.*

Next Generation Sequencing (NGS) was performed on a MiSeq sequencer (Illumina) by INRAE platform. The primers used target the ITS2 variable regions of genomic fungi. The ITS2 region was amplified from purified genomic DNA with the primers F (CTTTCCCTACACGACGCTCTTCCGATC-TTGTGARTCATCGAATCTTTG) and R (GGAGTTCAGACGTGTGCTCTTCCGATCT-TCCTCCGCTTATTGATATGC) using 35 amplification cycles with an annealing temperature of 55 °C. Because MiSeq enables paired 250-bp reads, the ends of each read are overlapped and can be stitched together to generate extremely high-quality, full-length reads of the entire region in a single run. Single multiplexing was performed using homemade 6 bp index, which was added to reverse primer during a second PCR with 12 cycles using forward primer (AATGATACGGCGACCACCGAGATCTACACTCTTTCCCTACACGAC) and reverse primer (CAAGCAGAAGACGGCATACGAGAT-index-GTGACTGGAGTTCAGACGTGT). The resulting PCR products were purified and loaded onto the Illumina MiSeq cartridge according to the manufacturer’s instructions. The quality of the run was checked internally using PhiX, and then each pair-end sequence was assigned to its sample with the help of the previously integrated index. The bioinformatics analysis was conducted using dada2 package v1.18.0 [34]. First, primers and chimeric reads were removed during filtering step. Then, sequences that showed 100% homology with each other were grouped into unique sequences and then into Operational Taxonomic Units (OTUs). Finally, OTUs were assigned with Unite database [32].

### 2.11. Sensitivity, Specificity, Positive Predictive Value, and Negative Predictive Value

Sensitivity is the probability of detecting the presence of a fungus, calculated as TP/(TP + FN). Specificity is the probability of rejecting an absent fungus, calculated as FN/(FP + TN). Positive predictive value (PPV = TP/(TP + FP)) indicates the probability that the pathogens are present when the test result is positive, whereas the negative predictive value (NPV = TN/(TN + FN)) is the probability that the fungi are absent when the test result is negative. Confidence interval was computed with the function proportion_confint using method beta from the Python package statsmodel; see [35]. *p*-value was calculated using McNemar’s chi2 from the R function Mcnemar test according to [36].

## 3. Results

### 3.1. Construction and Validation of the DendrisCHIP^®^DP for the Dermatophyte Diagnosis

A clinical overview supervised by the INOVIE Group (https://inovie.fr/), a major clinical laboratory network in France for medical diagnosis and supported by a recent epidemiological analysis of this infection in France [37], indicated that at least a set of 12 fungal pathogens could be considered as directly implicated in dermatophyte infections. Based on these data, we constructed the DendrisCHIP^®^DP, taking into account this panel of 12 species or genus of pathogens (Table 1). The development of this biochip required, at first, the design and the production of oligoprobes, which should be as specific as possible for each strain/species. The ribosomal internal transcribed sequence (ITS) regions of rDNA in the genome of these fungi were chosen as this region has been reported to be sufficiently polymorphic for species-level identification of dermatophytes [4]. The DendrisCHIP^®^DP was thus produced using a set of 46 oligoprobes designed on DendriSLIDE deposited in triplicates by Scienion’s sciFLEXARRAYER SX. 

After spotting the probes on the DendriSLIDE, the first step in validating the “DendrisCHIP^®^DP” was to ensure that the oligoprobes showed a positive hybridization signal with their corresponding DNA targets (see Figure S1 in Supplementary data). A multiplex PCR was performed with DendrisKIT^®^DP on the DNA of various pure strains of dermatophyte species. The purified amplicons were hybridized on the DendrisCHIP^®^DP using the DendriSTATION. After recording and annotating all these hybridization signals, a database was built up with a total of 441 samples (see details in Supplementary data Figure S2) from different sources where approximately 2/3 of the hybridization signals were obtained from pure microbial strains at different concentrations to mimic the abundance of the different pathogens in the samples. To integrate the matrix effect and, notably, the presence of possible PCR inhibitors, as well as the large amount of non-microorganism genomic DNA present in the overall extracted DNA, 1/3 of the database was constructed with clinical samples containing a well-identified pathogen, as well as with negative clinical samples spiked with a known pathogen. In addition, even if double dermatophyte infections are anecdotal [38], samples containing a mixture of two or three different fungal species were also included in this analysis to take into account the fact that a sample may contain more than one pathogen.

**Figure 1:**
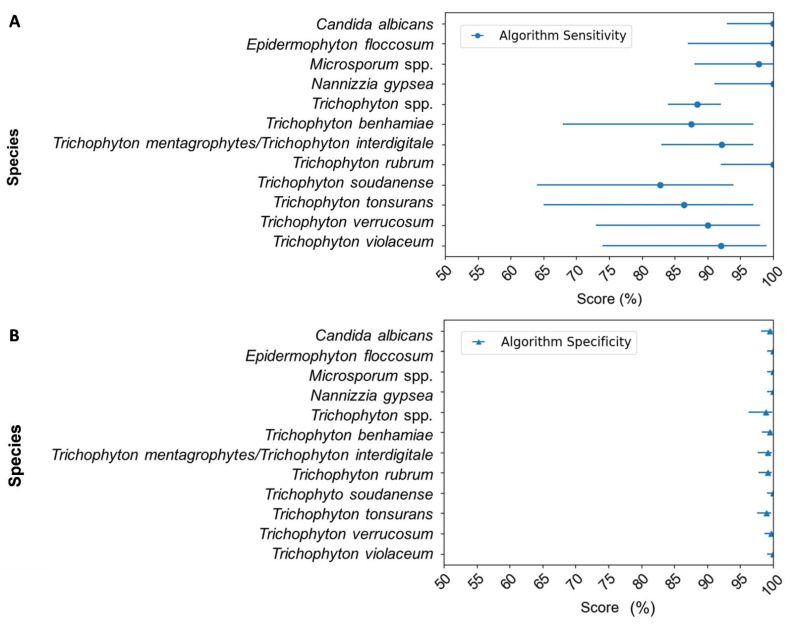
Performance of the DendrisCHIP^®^DP for each pathogen detected by the DendrisCHIP^®^DP on the database signals/data as described in Materials and Methods. (**A**) Sensitivity represented by points enclosed with 95% confidence interval. (**B**) Specificity represented by filled triangles with 95% confidence interval.

This database is then used as a training set to build a model, which enables the predictive value of the presence or absence of a pathogen in a sample. This machine learning method described in Section 2 is integrated into the DendriSOFT^®^ software (https://dendrisoft.fr/). After evaluating the model, we achieved 98% accuracy in detection, with sensitivity and specificity by pathogen reported as a forest plot in Figure 1. Using a dedicated program based on machine learning, it turned out that the discrimination of *Trichophyton mentagrophytes* and *Trichophyton interdigitale* was not possible, probably because these two species are phylogenetically too close, and they were therefore merged as *Trichophyton mentagrophytes/Trichophyton interdigitale*. Likewise, species belonging to the genus *Microsporum* were grouped under the name *Microsporum* spp. On the other hand, high sensitivity was obtained for *Candida albicans, Epidermophyton floccosum, and Nannizzia gypsea,* likely because the probes designed for these species were highly specific. A sensitivity of 100% [92–100%] was also noted for *Trichophyton rubrum,* probably due to the abundance of this pathogen in the clinical samples, providing a well-trained model for detecting this pathogen. In contrast, the sensitivity of *Trichophyton soudanense* and *Trichophyton tonsurans* was slightly lower, at 83% [64–94%] and 86% [65–97%], respectively. This lower sensitivity may be due to the paucity of clinical data on these species in the training database and to the high sequence similarity among the *Trichophyton* genus. Regarding specificity, all the values calculated were close to 100% (99% and 100%). Consequently, given that sensitivity for all pathogens was superior to 83%, we can conclude that the DendrisCHIP^®^DP has the potential to identify all 12 pathogens on the panel with high reliability.

We then assessed the limit of detection (LoD) of the DendrisCHIP^®^DP by hybridizing PCR amplicons obtained by serial dilutions of pure DNA from strains listed in Table 1. For most of the pathogens, a LoD in the range of 10–30 copies/µL was determined, except for *Trichophyton rubrum*, which was at 1.2 copies/µL, while a LoD of 100–200 copies/µL for *Candida albicans* and *Trichophyton mentagrophytes* was estimated (see Table S1 in Supplementary data). This low detection value could be explained by the lower specificity of the probes. Overall, the LoD determined with this DendrisCHIP^®^DP was of the same order as that previously reported for bacteria LoD using DendrisCHIP^®^ dedicated for respiratory and osteoarticular infection diseases [28,29]. Therefore, the similar LoD values, whatever the infection disease, suggest that the limit of detection is set by the technological features of the DendrisCHIP^®^ rather than by biological parameters.

### 3.2. More Positive Clinical Samples Detected by the DendrisCHIP^®^DP Than by Microbiological Culture

**Figure 2:**
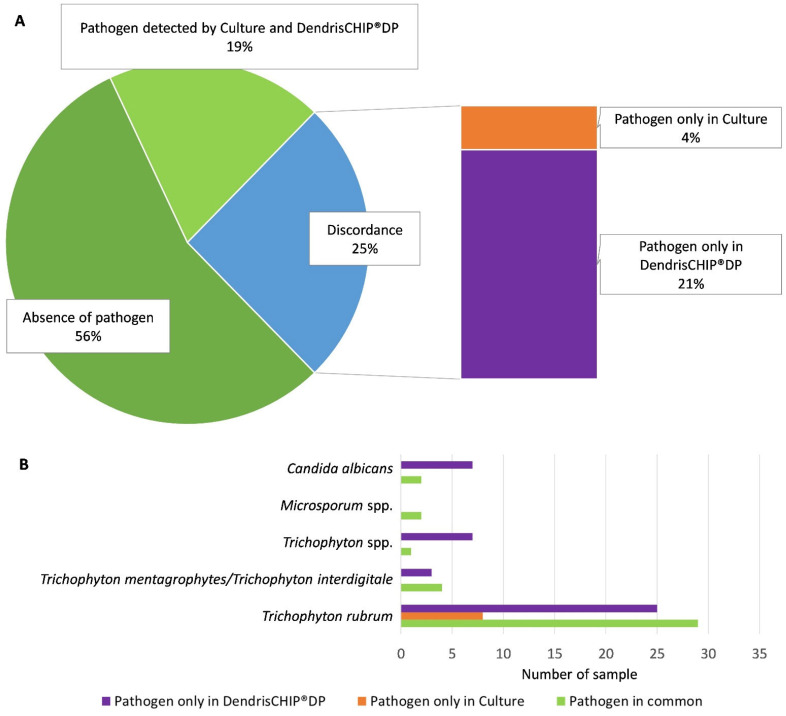
Comparison between DendrisCHIP^®^DP and conventional culture for the identification of pathogens in 197 residual clinical isolates. The distribution of concordant and discordant results with respect to the detection by culture is shown in panel (**A**). Panel (**B**) reports the number of isolates according to the pathogen species identified.

Skin scrapings, nail fragments, and hair samples were collected from patients with suspected dermatophyte infection. A total of 197 clinical isolates were divided into two parts, with one half to be analyzed by conventional microbiological culture carried out by clinical laboratories according to their internal protocol (see Section 2) and the other half to be processed through the DendrisCHIP^®^ technology. The pathogens identified by both methods were then compared. The results in Figure 2 showed a concordance of approximately 75% between the two methods. More precisely, the same species were found in 38 out of 197 samples, and in 109 samples, no pathogen was detected by either method. Note that of the 12 pathogens listed, only 5 were identified in all samples tested. More importantly, 42 out of 197 samples that were found to be negative by culture turned out to be positive with the DendrisCHIP^®^DP (at least for the presence of one pathogen). On the other hand, only 4% (8/197) of the samples that were negative with the DendrisCHIP^®^DP showed the presence of a pathogen with the microbiological method. Taking into account the pathogens identified in the samples, we saw that the most represented species was *Trichophyton rubrum*, followed *by Candida albicans*, *Trichophyton* spp., *Microsporum* spp., and *Trichophyton mentagrophytes/Trichophyton interdigitale*, which is very consistent with the species commonly found in dermatophyte infections in France [37].

In order to compare the conventional culture method with the DendrisCHIP^®^ technology, we had to split all solid samples into two parts, which can lead to a bias in the result due to the non-homogeneous distribution of pathogens within the samples. To overcome this potential bias, in addition to scraping, we sampled a further 87 patients by a direct superficial swabbing on the lesion (see Section 2). With this new sampling method, a concordance between the two methods of about 86% was recorded, with almost all pathogens found in culture also detected by DendrisCHIP^®^DP. Yet, 13% of the 87 samples turned out to be positive only with the DendrisCHIP^®^DP solution, whereas only 1% of pathogens were detected in culture and not by DendrisCHIP^®^DP. Also, the species distribution was similar to those obtained by the scraping sampling (Figure 2), with a large majority of *Trichophyton rubrum* (see Figure S3 in Supplementary data).

### 3.3. Dermatophyte Diagnosis by DendrisCHIP^®^DP Is More Reliable Than by Microbiology Culture 

**Figure 3:**
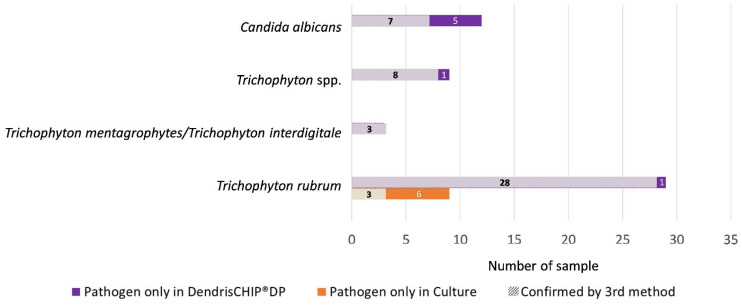
Validation by a third molecular method (RT-PCR and NGS) of discordant results between DendrisCHIP^®^DP and conventional culture for the identification of pathogens in clinical isolates suspected of dermatophytosis. The number of isolates according to the pathogen species identified is reported.

The data reported above suggest a higher reliability of DendrisCHIP^®^DP with respect to the culture method in terms of pathogen detection and species identification. To support this assertion, the 62 samples (50 residuals and 12 swab samples) that showed discordant results between the two methods were analyzed by a third method using either RT-PCR or NGS techniques. As shown in Figure 3, 46 out of 53 (86%) positive samples identified by DendrisCHIP^®^DP were confirmed by a third method, whereas only 3 out of the 9 positive samples identified only by the culture method were confirmed by a third method. These data confirmed that DendrisCHIP^®^DP is more reliable in identifying dermatophytes in isolates than the culture method. 

**Figure 4:**
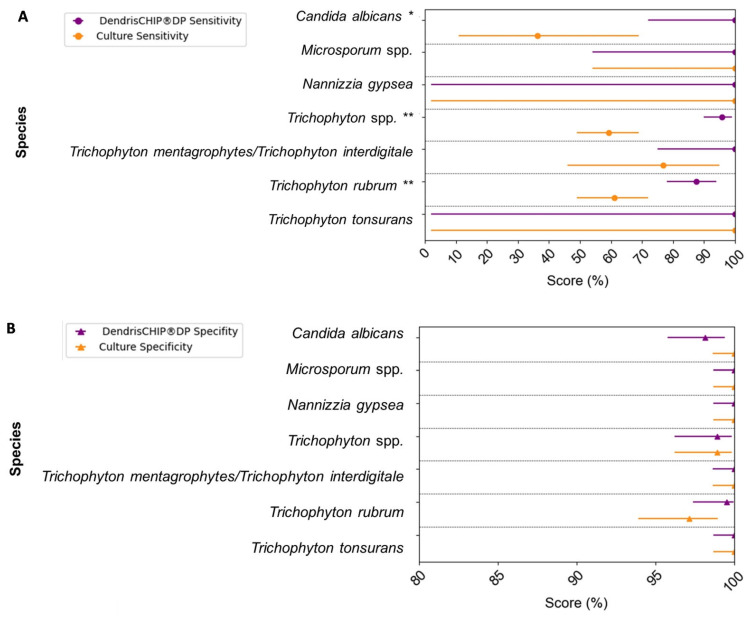
Comparison between DendrisCHIP^®^DP and conventional culture for the identification of pathogens in a total of 284 clinical isolates. (**A**) Sensitivity represented by points enclosed with 95% confidence interval in purple for DendrisCHIP^®^DP and in orange for culture. (**B**) Specificity represented by triangle enclosed by a 95% confidence interval in purple for DendrisCHIP^®^DP and in orange for culture. * Significant difference between DendrisCHIP^®^DP and culture with *p*-value < 0.05 and ** *p*-value < 0.01.

Collectively, all these data enabled us to calculate sensitivity, specificity, PPV, and NPV according to Safari et al. [39], as detailed in Section 2.11 of Section 2. As shown in Figure 4, the sensitivity was globally better with DendrisCHIP^®^DP than with microbial culture, whereas specificity was roughly similar for both methods. On the other hand, calculations of PPV and NPV gave rise to the same trend, whatever the methodology used for the diagnostic (see Table S2 in Supplementary data). The higher sensitivity of the DendrisCHIP^®^DP compared to the third technique, particularly for *Candida albicans*, could lead to an overestimation of false positives and an underestimation of PPV for DendrisCHIP^®^DP. Taken together, these results indicate that the DendrisCHIP^®^DP turned out to be more reliable in detecting and identifying pathogens in clinical samples than the microbiological culture. In addition, this molecular technology is faster than the culture, as the results can be obtained within 48 h upon receipt of the patient sample.

## 4. Discussion

Dermatophytes are the most common cause of fungal infections worldwide, affecting millions of people each year. Since Sabouraud in 1904, the diagnosis of dermatophytosis has largely been based on a combination of microscopy and microbiological culture. Clinical mycologists are looking for a method that is not only faster but also has higher throughput analysis capabilities to improve the therapeutic management of an ever-increasing number of patients [40]. In addition, it is imperative that this diagnostic technology becomes easy to use routinely and is cost-effective. To address these challenges, we proposed a molecular-based method, the DendrisCHIP^®^ technology, that is rapid, robust, and automated, which was evaluated for respiratory and osteoarticular infections [28,29].

In the present study, we wanted to apply the DendrisCHIP^®^ technology to the diagnosis field of dermatophytes and compare the efficiency of this method with classical mycological cultures. This comparative analysis carried out on a total of 284 suspected case samples revealed that while the concordance between the two methods is around 75%, 21% of the samples that gave a negative result with the culture method were, in fact, positive with the DendrisCHIP^®^DP and this presence of pathogen was confirmed at 86% by RT-PCR or sequencing. This study convincingly shows that DendrisCHIP^®^DP outperforms the conventional diagnostic method in its ability to detect and identify pathogens present in clinical isolates from patients with dermatophyte infections. In agreement with other studies [41,42,43], our results demonstrate that molecular PCR techniques based on the use of appropriate DNA sequences are far more relevant than conventional microscopy and culture methods for dermatophytosis diagnosis.

Overall, the greater efficiency of our technique can be explained by the fact that DNA detection avoids the limitations of the phenotypic methods. Indeed, culture has a high false negative rate because the proliferation of non-dermatophyte molds in the culture medium may prevent or mask the development of a pathogen [44] or that previous antifungal treatment has resulted in non-viable fungi unable to grow in a culture which is not limiting for PCR detection [45,46]. Moreover, the difference between results may be explained by the inclusion of fungi in keratin [19] and the fact that the scraping samples were divided into two parts, each part not necessarily homogeneous in terms of fungal element distribution [38]. We therefore tested this potential bias using an additional swab sampling procedure on 87 samples. The discrepancy level was reduced from 25% to 14%, with DendrisCHIP^®^DP detection failing in less than 1% of cases. 

The advantages of this technology over PCR-based technologies for diagnostics in general, and for fungal infections in particular, are (i) higher multiplexing expressed in terms of the number of pathogens that can be detected per sample, (ii) rapid diagnostics made possible by the use of artificial intelligence tools, and (iii) high-throughput capabilities thanks to semi-automation enabling the analysis of 192 samples per day. In addition, the implementation of this technology does not require any particular expertise and is easily accessible to clinical laboratory technicians. The combination of these advantages makes the cost of analysis per sample much more competitive than that of PCR methods. Overall, dermatophytosis diagnosis using DendrisCHIP^®^ technology offers exactly the same advantages in terms of time scale and readiness as it is described in the workflow for the diagnosis of osteoarticular diseases [29].

Taken together, these results demonstrate excellent performance characteristics for the detection of dermatophytes by the DendrisCHIP^®^DP, making it very promising for routine dermatophytosis diagnostics. It can help the clinicians to initiate timely and appropriate antifungal therapy. The future steps will be to enhance the performance of DendrisCHIP^®^DP by increasing its fungal pathogen panel, improving detection for some of these pathogens, and providing clinical evaluations of this new technology through an independent multi-center study involving academic and private laboratories. 

## Figures and Tables

**Table 1:** List of pathogens targeted by the DendrisCHIP^®^DP.

Fungal Microorganisms	Taxonomy	Source	Accession Number
*Candida albicans*	species	Vircell ATCC 90028	LC388876.1
*Epidermophyton floccosum*	species	CECT 2769	LC317573.1
*Microsporum* spp.	genus	CHU Purpan	AJ006251.1/LC53030.1
*Nannizzia gypsea*	species	BCCM/IHEM 25157	LC170561.1
*Trichophyton* spp.	genus	CHU Purpan	-
*Trichophyton benhamiae*	species	CECT 2892	LT897802.1
*Trichophyton mentagrophytes/Trichophyton interdigitale*	species	CECT 2901/CECT 2793	LC317813.1/LC413778.1
*Trichophyton rubrum*	species	CECT 2794	LC404119.1
*Trichophyton soudanense*	species	BCCM/IHEM 20772	MF173063.1
*Trichophyton tonsurans*	species	BCCM/IHEM 24955	AB220044.1
*Trichophyton verrucosum*	species	CECT 2992	AB491473.1
*Trichophyton violaceum*	species	BCCM/IHEM 26519	AB430482.1

## Data Availability

Data are contained within the article and supplementary materials.

## Supplementary Materials

The following supporting information can be downloaded at https://www.mdpi.com/article/10.3390/diagnostics13223430/s1. Figure S1: Intensity of oligoprobes designed for each pathogen on DendrisCHIP^®^DP expressed as boxplots. The figure shows the oligoprobes enabling the identification of pathogens by our decision algorithm; Table S1: List of pathogens targeted by the DendrisCHIP^®^DP with the limit of detection accessed as described in Materials and Methods. Figure S2: Repartition of 441 samples in the training database by pathogen, with the different types of samples colored in red for clinical samples, green for pure strains, blue for mixtures of strains, and purple for spikes; Figure S3: Comparison between DendrisCHIP^®^DP and conventional culture for the identification of pathogens in clinical samples of swabs. The distribution of concordant and discordant results with respect to the detection by culture is shown in panel A. Panel B reports the number of isolates according to the pathogen species identified; Table S2: Evaluation of the predictive positive and negative values (PPV; NPV) of diagnostic tests by DendrisCHIP^®^DP and conventional culture relative to data obtained from 284 confirmed isolates (with 95% confidence interval indicated in brackets).
