# Supplementary material for: Rapid and Accurate Diagnosis of Dermatophyte Infections Using the DendrisCHIP® Technology
Source: Diagnostics (Basel). 2023 Nov 11;13(22):3430. doi: 10.3390/diagnostics13223430 (PMC10670032; doi:10.3390/diagnostics13223430)
Supplement: Supplementary file 1 [file diagnostics-13-03430-s001.zip › diagnostics-2697121-supplementary.pdf]

Supplementary Materials: Rapid and accurate diagnosis of dermatophyte infections using the DendrisCHIP® technology

Aurore Anton[1 *]; Mathilde Plinet[1], Thomas Peyret[1], Thomas Cazaudarré[1], Stéphanie Pesant[1], Yannick Rouquet[2], Marie-Andrée Tricoteaux[2], Matthieu Bernier[2], Jérémy Bayette[3], Remi Fournier[3], Mélanie Marguerettaz[3], Pierre Rolland[3], Thibaud Bayol[3], Nadia Abbaoui[3], Antoine Berry[4], Xavier Iriart[4], Sophie Cassaing[4], Pamela Chauvin[4], Elodie Bernard[1], Richard Fabre[1] & Jean-Marie François[1,5]

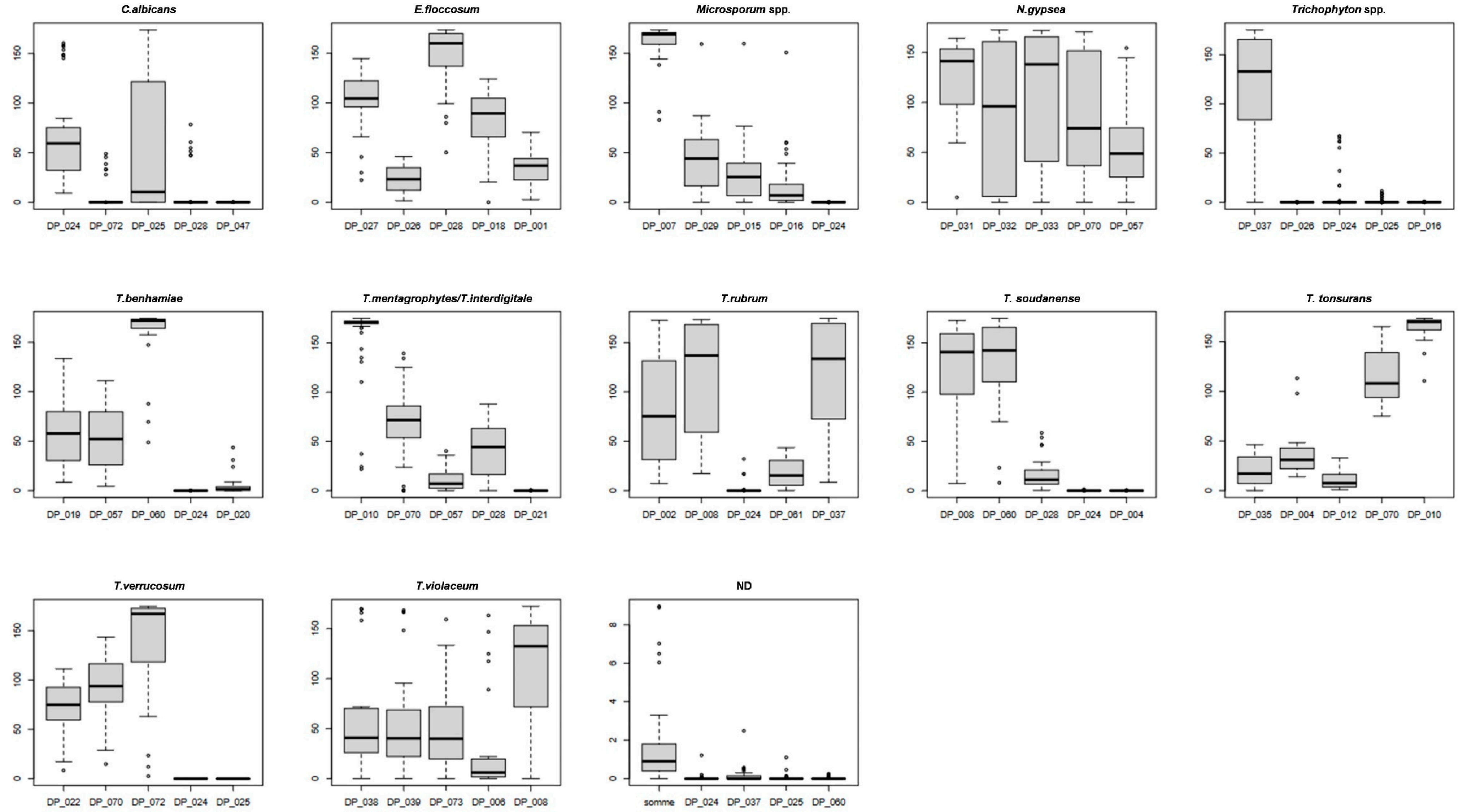

Figure S1. Intensity of oligoprobes designed for each pathogen on DendrisCHIP®DP expressed as boxplots. The figure shows the oligoprobes enabling the identification of pathogens by our decision algorithm.

Fungal Microorganisms	Limit of detection (copies/µL)
Candida albicans	125
Epidermophyton floccosum	19
Microsporum spp.	-
Nannizzia gypsea	11,9
Trichophyton spp.	-
Trichophyton benhamiae	22
Trichophyton mentagrophytes/Trichophyton interdigitale	210/200
Trichophyton rubrum	1,2
Trichophyton soudanense	33
Trichophyton tonsurans	27
Trichophyton verrucosum	20
Trichophyton violaceum	20

Table S1. List of pathogens targeted by the DendrisCHIP®DP with the limit of detection accessed as described in Materials and Methods.

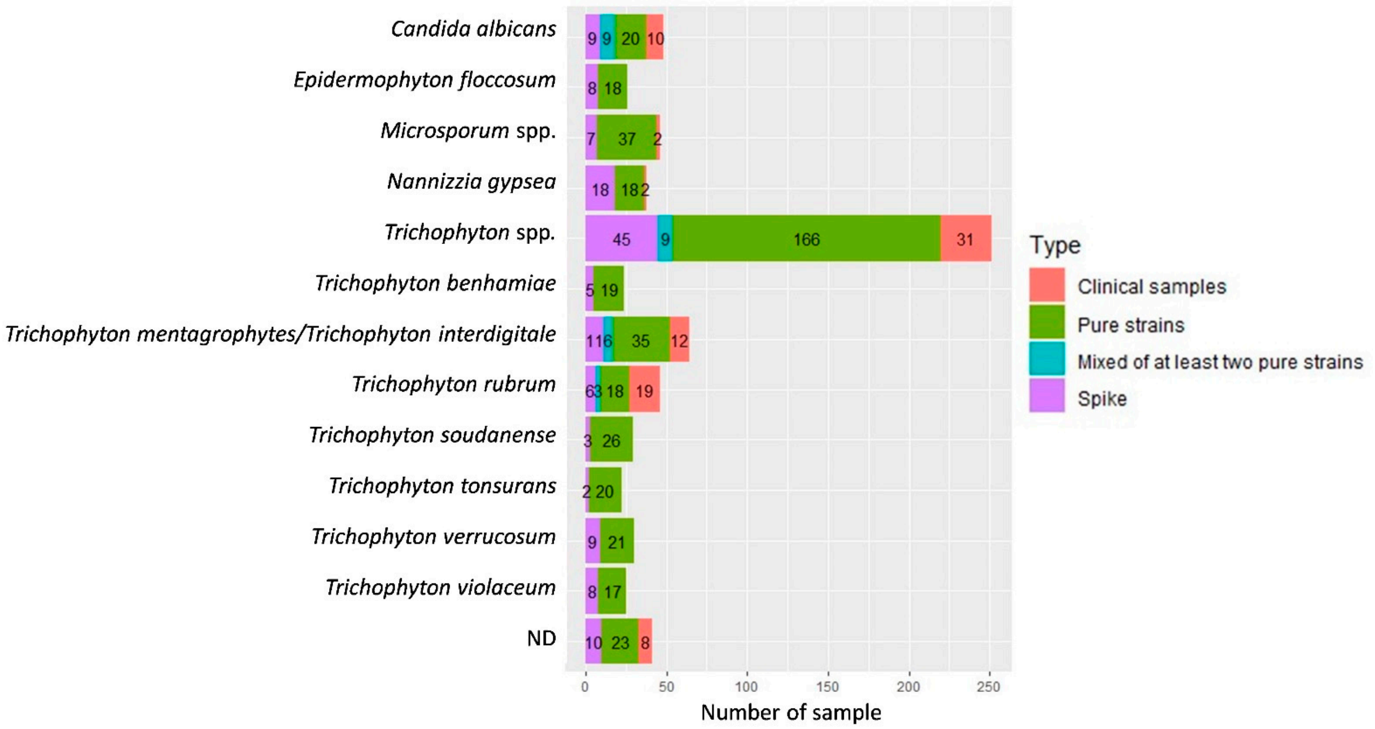

Figure S2. Repartition of 441 samples in the training database by pathogen, with the different types of samples colored in: red for clinical samples, green for pure strains, blue for mixtures of strains and purple for spikes.

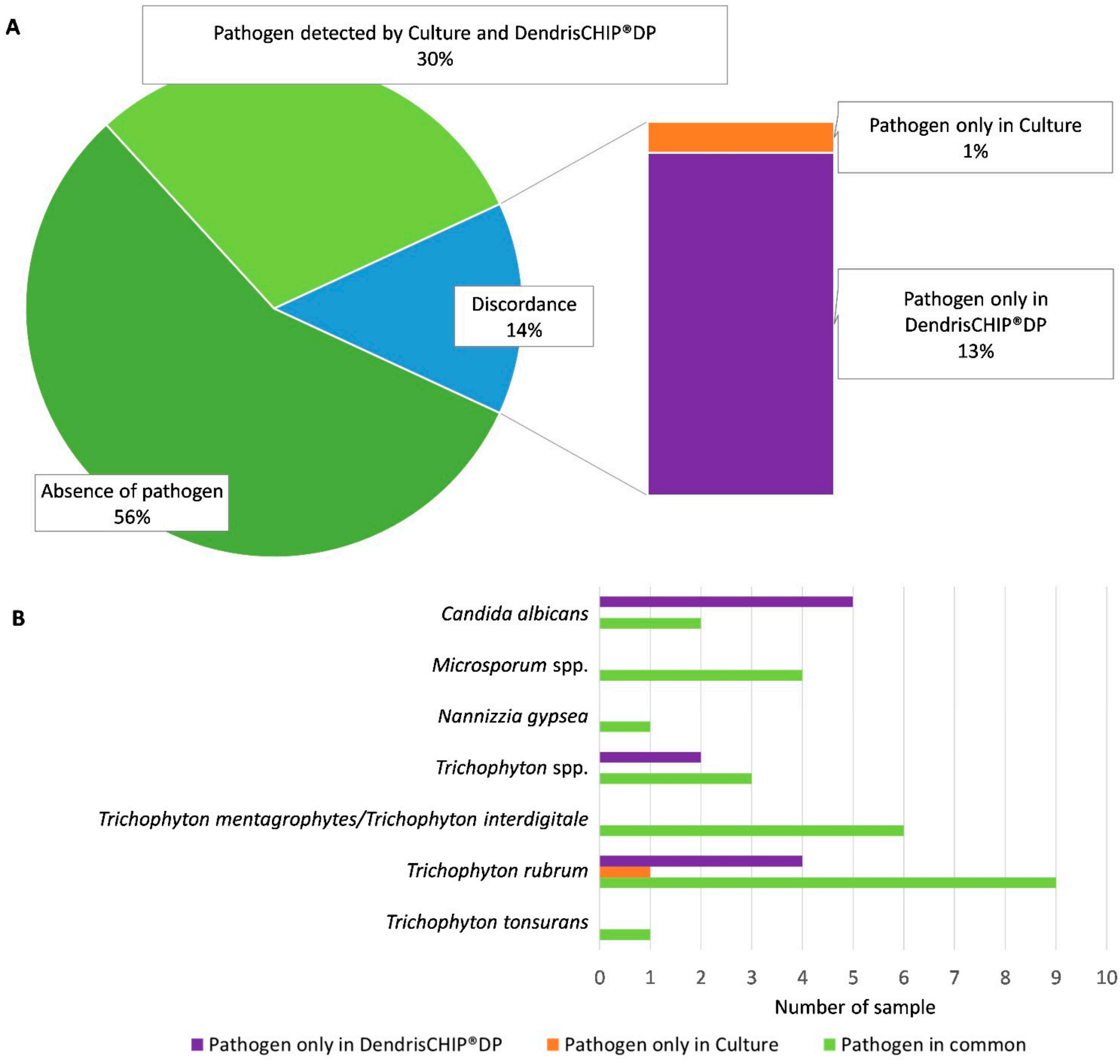

Figure S3. Comparison between DendrisCHIP®DP and conventional culture for the identification of pathogens in swabs clinical samples. The distribution of concordant and discordant results with respect to the detection by culture is shown in panel **A**. In panel **B** is reported the number of isolates according to the pathogen species identified.

	PPV (CI 95)		NPV (CI 95)	
	Dendris	Culture	Dendris	Culture
Candida albicans	68,8% (41-89)	100% (40-100)	100% (99-100)	97,5% (95-99)
Microsporum spp.	100% (54-100)	100% (54-100)	100% (99-100)	100% (99-100)
Nannizzia gypsea	100% (2,5-100)	100% (2,5-100)	100% (2,5-100)	100% (2,5-100)
Trichophyton spp.	97,9% (93-100)	96,6% (88-100)	97,9% (95-99)	82,7% (77-87)
Trichophyton rubrum	98,4% (92-100)	88,0% (76-95)	95,9% (76-95)	88,0% (83-92)
Trichophyton mentagrophytes/Trichophyton interdigitale	100% (75-100)	100% (69-100)	100% (99-100)	98,9% (97-100)
Trichophyton tonsurans	100% (2,5-100)	100% (2,5-100)	100% (2,5-100)	100% (2,5-100)

Table S2. Evaluation of the predictive positive and negative values (PPV; NPV) of diagnostic tests by DendrisCHIP®DP and conventional culture relative to data obtained from 284 confirmed isolates (with 95% confidence interval indicated in brackets).
